# The invention of writing on Rapa Nui (Easter Island). New radiocarbon dates on the Rongorongo script

**DOI:** 10.1038/s41598-024-53063-7

**Published:** 2024-02-02

**Authors:** Silvia Ferrara, Laura Tassoni, Bernd Kromer, Lukas Wacker, Michael Friedrich, Francesca Tonini, Lorenzo Lastilla, Roberta Ravanelli, Sahra Talamo

**Affiliations:** 1 Department of Classical Philology and Italian Studies, Alma Mater Studiorum, University of Bologna, Via Zamboni 32, 40126 Bologna, Italy; 2 Department of Chemistry G. Ciamician, Alma Mater Studiorum, University of Bologna, Via Selmi 2, 40126 Bologna, Italy; 3 Institute for Environmental Physics, University of Heidelberg, 69120 Heidelberg, Germany; 4 Laboratory for Ion Beam Physics, ETH Zurich, 8093 Zurich, Switzerland; 5 Hohenheim Gardens, University of Hohenheim, 70599 Stuttgart, Germany; 6 Alma Mater Studiorum, Università di Bologna-Ravenna Scientific Campus SCoRe, 48121 Ravenna, Italy; 7 Department of Computer, Control and Management Engineering Antonio Ruberti (DIAG), Sapienza University of Rome, 00185 Rome, Italy; 8 Department of Civil, Constructional and Environmental Engineering (DICEA), Sapienza University of Rome, 00184 Rome, Italy

**Keywords:** Anthropology, Archaeology, Cultural evolution

## Abstract

Placing the origin of an undeciphered script in time is crucial to understanding the invention of writing in human history. Rapa Nui, also known as Easter Island, developed a script, now engraved on fewer than 30 wooden objects, which is still undeciphered. Its origins are also obscure. Central to this issue is whether the script was invented before European travelers reached the island in the eighteenth century AD. Hence direct radiocarbon dating of the wood plays a fundamental role. Until now, only two tablets were directly dated, placing them in the nineteenth c. AD, which does not solve the question of independent invention. Here we radiocarbon-dated four Rongorongo tablets preserved in Rome, Italy. One specimen yielded a unique and secure mid-fifteenth c. date, while the others fall within the nineteenth c. AD. Our results suggest that the use of the script could be placed to a horizon that predates the arrival of external influence.

## Introduction

Rapa Nui, also known as Easter Island, is located in the deepest recesses of the Pacific Ocean, some 3800 km off the coast of Chile. It was one of the latest landmasses to be settled by humans, according to radiocarbon dating, between 1150 and 1280 AD^1^. Since then, the island underwent a gradual, if not total, process of deforestation^2^. When Rapa Nui was discovered by Europeans seafarers in the 1720s, its soil had been to an extent eroded and depleted, and endemic species, once luxuriant, had disappeared, even though this ecocide picture of total devastation is now under serious scholarly debate^3,4^. The arrival of European visitors, in any case, brought upheaval. Sporadic raiding and kidnapping of locals took place in the early 1800s, and later during that century Peruvian slave raids were carried out, while epidemics decimated the population^5^. By the end of the century, most of its traditional culture was irretrievably lost.

**Figure 1:**
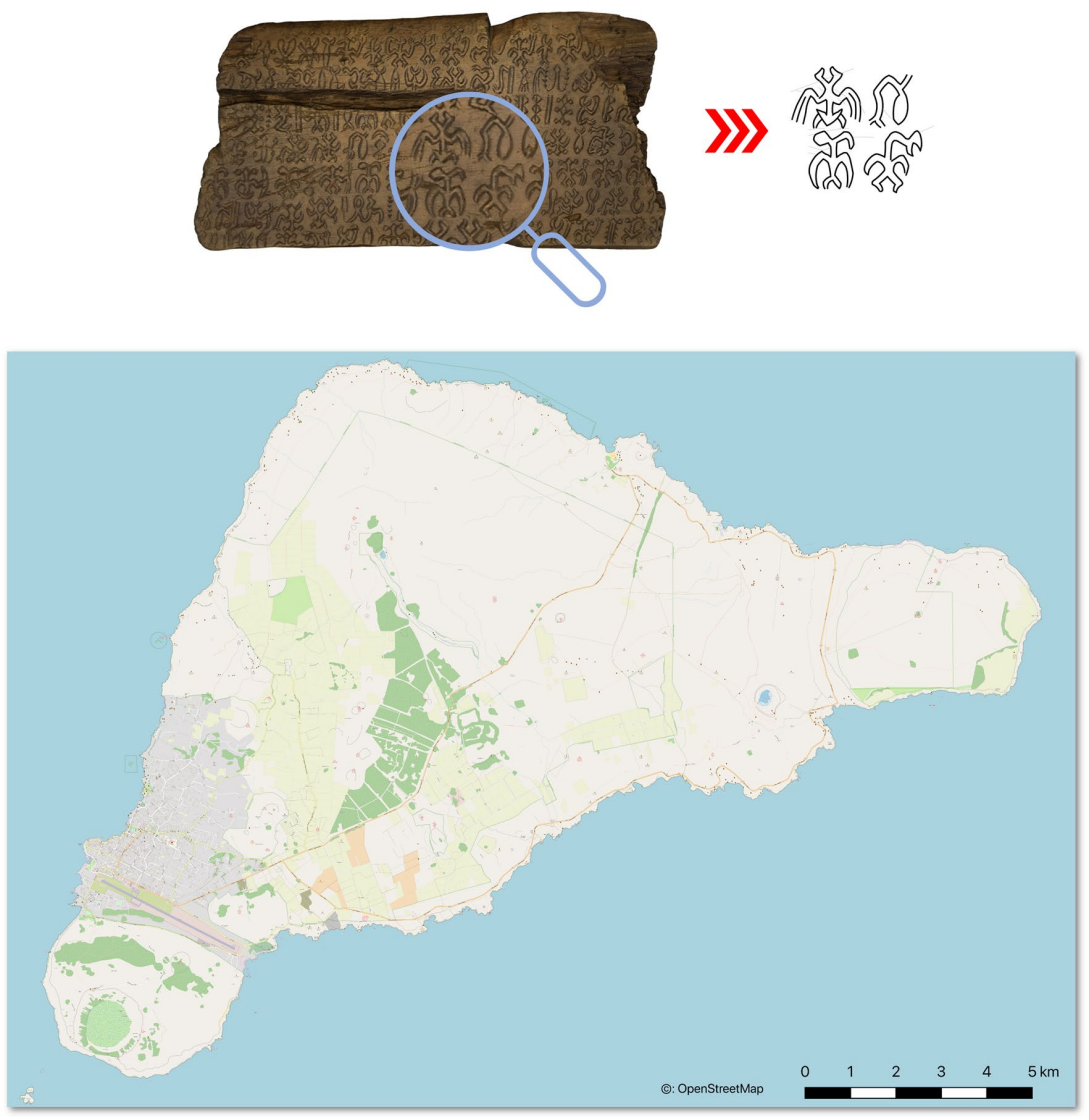
Rapa Nui tablet and location. (**A**) 3D model of the Rapa Nui tablet D Échancrée. (**B**) Enlargement on the script. (**C**) Map of Rapa Nui (produced with OpenStreetMap, available under the Open Database License).

Writing is one of the local phenomena to fall prey to destruction. While the island is famous for its monumental sculptures, called *moai* and still preserved in situ, its inhabitants also developed a local script, Rongorongo, which was first noticed by outsiders in 1864. The script now survives on twenty-seven wooden objects^6^ none of which is now on the island. Most were salvaged by missionaries in the 1860s and 1870s and sent abroad. Not all rescue attempts were successful, and some inscriptions were intentionally destroyed. The extant texts are relatively long and written by means of pictorial signs, often called ‘glyphs’ (Fig. 1).

Discovering a writing system in such a remote recess is surprising, and debate is still ongoing as to its origins. While it is difficult to prove that contact with literate Europeans was not a stimulus for its creation, its pictorial glyphs do not resemble any known script. They, in fact, show their closest parallels in motifs of ancient rock-carved art found on the island^7^. The shapes of the Rongorongo signs represent different classes of images, such as human postures and body parts, animals, plants, tools, heavenly bodies, etc. The use of these signs in complex ligatures and long, linear sequences, and evidence of corrections^8^, suggest proper language notation.

Despite this, the script today remains undeciphered since the tablets ceased to be produced in the late 1860s, after the raids^6^. If the date for its demise seems thus clear, the exact period in which Rongorongo developed remains unknown. The question is of crucial importance, as it implies the possibility of an independent invention of writing, similarly to what happened in other parts of the world where writing was an original creation, e.g., in Mesopotamia, Egypt, China and Mesoamerica. If Rongorongo predates the arrival of external travelers, it could represent another, and the latest, invention of writing in human history.

To cast light on this issue, direct radiocarbon analysis plays a fundamental role. Until now, only two tablets of the Rongorongo corpus have been dated. Tablet Q, housed in the Museum of Anthropology and Ethnology in St. Petersburg (and called the Small St. Petersburg tablet), was dated in 2003^8^ and produced three ranges: 1680–1740 AD, 1800–1930 AD, and 1950–1960 AD (conventional radiocarbon date AD 1950 = 0 BP, before present). Recently it was recalibrated using the Southern Hemisphere atmospheric curve SHCal20^9^, and the most reliable date appears to be 1812–1836 AD^6^. Tablet O, housed in the Ethnological Museum Dahlem in Berlin, yielded a calibrated range of 1811–1838 AD^10^. In 1770 AD a ceremony is recorded where the islanders were observed writing and practicing with a quill, and the probability of obtaining a large branch of wood seems more likely in the first half of the eighteenth c. as opposed to the first half of the nineteenth. In any case, these dates are only one partial achievement and crucially do not allow to reach any incontrovertible conclusion as to whether Rongorongo was the product of contact with external inputs or an original invention. To add substantial evidence to either hypothesis, more inscribed objects need to be radiocarbon dated, and this is the reason why we deemed it essential to add further analysis.

To this end, we examined four new wooden objects inscribed in Rongorongo housed at the Congregazione dei Sacri Cuori di Gesù e di Maria, in Rome, Italy. All four objects were corralled by European missionaries in 1869 and sent to the Bishop of Tahiti, Tepano Jaussen. They were subsequently sent back to Europe at the end of the nineteenth c. AD. In the standard corpora they are cataloged as texts A-D (see Supplementary Note 2 and Supplementary Table 1).

Tablet A is called Tahua (P001) and presents a long text on an object shaped as an oar blade. This tablet measures 91.2 × 11.5 × 2.8 cm in size and is made of *Fraxinus excelsior*^5^ which is native to Europe and never grew on the island. Tablet B, called Aruku Kurenga (P002), presents a dozen lines of text on both sides, is masterfully carved, and it measures 41.5 × 15.2 × 3.1 cm. Its wood is Pacific rosewood (*Thespesia populnea*), which once grew on the island. Tablet C, the Mamari (P004), measures 29.0 × 19.4 × 2.3 cm, and is neatly inscribed on both sides. The wood is again *Thespesia populnea*, and in some areas, it is damaged. The inscription was engraved after the damage to the surface, as some signs are carved inside the cavity. Tablet D or Échancrée (P003), measures 23.9 × 12.3 × 2.4 cm. The wood is identified as *Podocarpus latifolia*^11^, which is native to southeastern Africa and never grew on the island. It has been suggested that the two sides of this tablet may have been engraved by two different scribes^5^, one side smoothed and well-engraved, the other rougher and less elaborate, also in handwriting. This implies a two-step composition for the text. After completion, notches were cut into the long sides of the tablet, which was reused as a winding spool for a cord.

The botanical analysis is important to contextualize the inscriptions historically. The wood of tablet B and C, *Thespesia populnea*, is characterized by a powerful symbolism. The tree is tall and grows in eastern Polynesia on the shores of atolls and high islands^12^. It may have been brought to Rapa Nui by the first settlers^13^. It became wood par excellence: the generic old Rapa Nui word for ‘timber’ and wooden vessels is *miro*, whose cognates in all East Polynesian languages name the *Thespesia populnea (POLLEX, *https://pollex.eva.mpg.de/entry/milo1/*)*. Particularly malleable and easy to polish, its trees were planted close to the *ahu moai*, the platforms on which the famous *moai* statues stood*.* The choice of this wood to carve artifacts may owe as much to symbolic aspects as accessibility criteria^2^. The wood of tablets A and D, *Fraxinus excelsior* and *Podocarpus latifolia*, respectively, reached the island from elsewhere (perhaps Europe and southern Africa, respectively). The wood of D, *Podocarpus latifolia*, was also used for other tablets in the Rongorongo corpus, the Small Vienna tablet, the Large St. Petersburg tablet, and the Large Washington tablet (respectively labeled as N, P, and S). This type of conifer wood appears unlikely to have reached the island on four different occasions. Orliac^12^, suggested that they may all be part of the same large board, and the measurements and shapes of N, P and S suggest that this is true for at least these three items. Thus, tablets N, P, and S could be contemporaneous with the Échancrée (tablet D).

**Figure 2:**
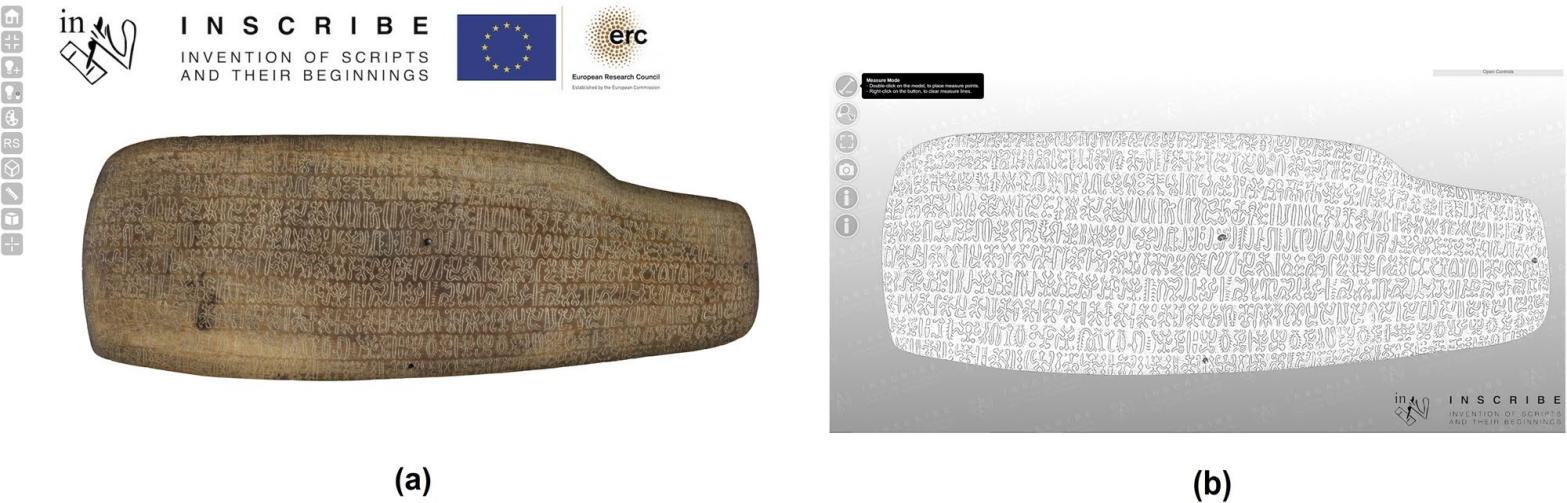
3D model of tablet B Aruku Kurenga (sample P002), with a high quality texture (**a**) and a high resolution geometry (**b**), as presented on the INSCRIBE 3D Interactive Web Viewer (www.inscribercproject.com/3d_viewer_home.php).

In addition, to present our evidence in the most unbiased, complete, and interactive way possible, we produced 3D models of the four objects housed in Rome (Fig. 2) before extracting the samples, to digitally preserve the pre-extraction status of the tablets and to design the optimal sampling locations on the objects. The models are available online on the INSCRIBE 3D Interactive Web Viewer (www.inscribercproject.com/3d_viewer_home.php). Three-dimensional digital models guarantee accurate visual investigations, measuring, and virtual manipulation, to assess the geometry, topology, and physical properties of the objects, and to correct the extant text transcriptions. Also, Rongorongo is a three-dimensional writing system, as the surfaces of the wooden tablets are curved and the glyphs themselves, finely carved into these surfaces, are not flat^14^. And finally, the models allowed us to create such a defined replica of the objects that we were able to assess with the utmost precision where to extract the samples.

To ensure correct dating, cellulose as a product of an efficient extraction protocol, free from any external carbon contamination, is fundamental. We sampled the 4 Rongorongo tablets (Supplementary Fig. 1 and Table 1), stored at the Congregazione dei Sacri Cuori di Gesù e di Maria in Rome. We used the BABAB (Base–Acid–Base–Acid–Bleaching) pretreatment protocol published by Cercatillo et al.^15^. The radiocarbon dates of the four different tablets suggested that three of them were of wood of the same period, and one (BRA-6127, sample P003 tablet D, Échancrée) was made with older wood (Table 1 and Supplementary Note 3). To date the tablets accurately we estimated the felling year of the tree from which the tablet was made, by taking into account the temporal distance from the position of the AMS sample to the outermost annual ring and considered the differences in the ^14^C-calibration age ranges (Supplementary Note 4 and Supplementary Table 2).

**Table 1:** Radiocarbon ages and the cellulose obtained for the four tablets.

Sample number in Rome	Bologna pretreatment code	Start mass (mg)	Cellulose mass (mg)	%cell	Bologna graphite code	AMS code	^14^C age	Error 1σ
(Tahua) P001	BRA-6125	46.3	25.2	54	B.Age 215	ETH-125842.1.1	122	13
(Aruku Kurenga) P002	BRA-6126	40.2	14.4	36	B.Age 216	ETH-125843.1.1	125	13
(Échancrée) P003	BRA-6127	74.4	35.1	47	B.Age 217	ETH-125844.1.1	474	13
(Mamari) P004	BRA-6129	48	28.9	60	B.Age 218	ETH-125845.1.1	189	13

We calibrated the samples using the South Hemisphere calibration curve SHCal20 in OxCal 4.4 program^9,16^. The calibration ranges discussed are the one considering the felling date of the tree, including an estimated error of 5 years (Supplementary Table 2). All calibrated intervals are discussed based on the 68.3% probability range.

The result of sample P001 tablet A Tahua (BRA-6125), including 50 ± 5 years to the felling date, is between 1862 and 1977 cal AD, this probability range has two main % probabilities: the main one (48.6%) between 1934 and 1977 cal AD, and one at 19.7% between 1862 and 1887 cal AD (Supplementary Fig. 2). Despite the higher percentage, the range falling to 1934–1977 cal AD should be discarded, as it falls after the date of collection and rescue of the Rongorongo tablets (1869) and falls after the Peruvian slave raids and the subsequent decline of Rongorongo. So, despite the higher probability (48.6%), this range cannot be accepted. The most prominent range is therefore the earlier one (1862–1887 cal AD), which pre-dates the raids and obsolescence of the script. Noteworthy are the properties of the tablet (good preservation, with no apparent damage, calligraphy).

The result of sample P002 tablet B Aruku Kurenga (BRA-6126), including 50 ± 5 years to the felling date, ranges between 1732 and 1947 cal AD, this range produced three different percentage probabilities once calibrated. One at 45.5% between 1903 and 1947 cal AD, one at 20.7% between 1832 and 1857 and the lowest one at 2.1% between 1732 and 1736 cal AD (Supplementary Fig. 3). Tablet B Aruku Kurenga is in a similar predicament as tablet A Tahua. The later range is to be discarded, even though it shows the highest probability. More prominent is the range 1832–1857 cal AD, which shows that the tablet must have been engraved soon after the arrival of European travelers. The smallest percentage pushes the date back to the eighteenth century, before contact with external influxes.

Sample number P003 tablet D Échancrée (BRA-6127), including 50 ± 5 years to the felling date, yields an older age compared to the other samples, ranging from 1493 to 1509 cal AD. Due to the absence of plateaus on the calibration curve, and the small ^14^C error range, the calendar ranges are more precise and constrained (Supplementary Fig. 4). Such an early age was not expected, and it shows that the date of the wood was some centuries earlier than the arrival of the Europeans in the 1720s.

The sample P004 tablet C Mamari (BRA-6129), including 50 ± 5 years to the felling date, ranges between 1694 and 1840 cal AD, and has four different probabilities: one at 32.4% between 1694 and 1727 cal AD, one at 18.2% between 1744 and 1770 cal AD, one at 5.3% between 1819 and 1840 cal AD, and the very small one at 2.4% between 1781 and 1789 cal AD (Supplementary Fig. 5). Three ranges of the four expand the range of tablets A and B, reaching back to the late seventeenth c. AD. The most prominent date places us with a high degree of probability in that century, while the other ranges in the eighteenth c., and beginning of the nineteenth c. fit well with the dates of tablets A, B, O and Q.

**Figure 3:**
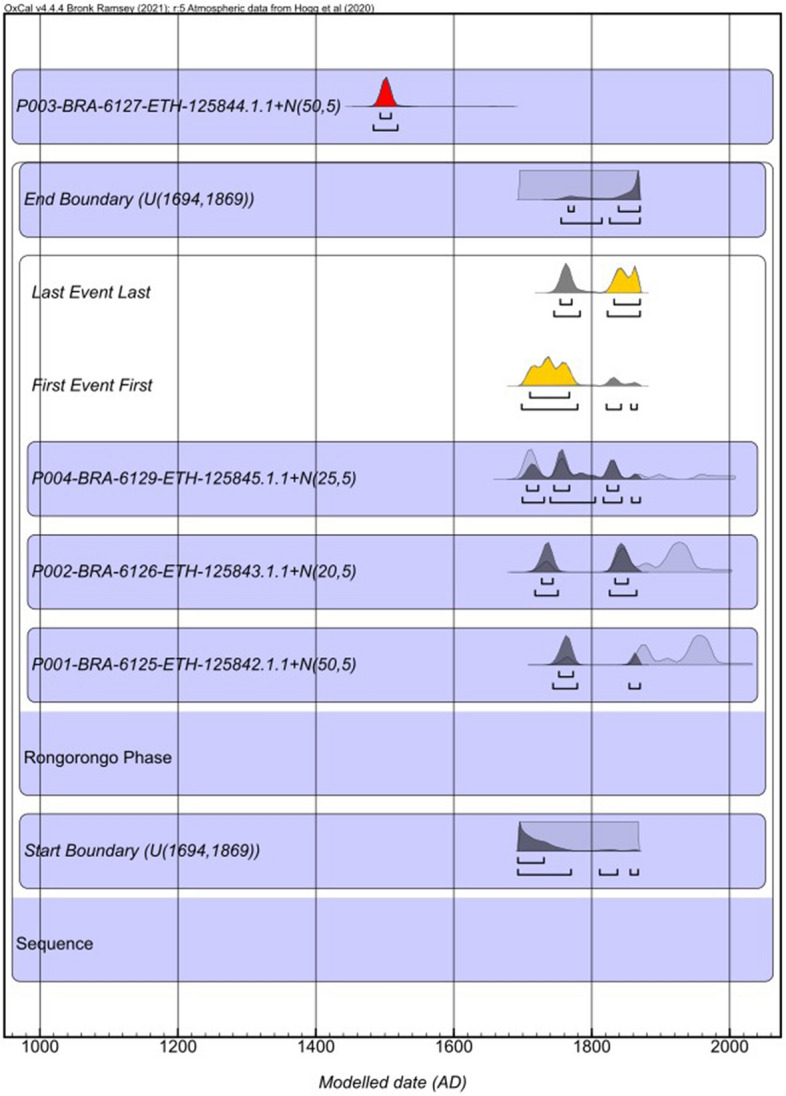
Bayesian model. Bayesian model of three tablets (BRA-6125; 6126; 6129) and the calibrated age of the BRA 6127 in red. Radiocarbon dates were calibrated and modeled using SHcal2011 in the OxCal 4.4 program^16^.

To better constrain the calendar ages of the Rongorongo tablets we constructed a Bayesian model using the OxCal 4.4 program18. The model (Fig. 3 and Table 2) is made in one single phase (Rongorongo Phase) adding only our 3 ^14^C results spanning almost the same period. The oldest date of BRA-6127 is inserted outside the Bayesian model to obtain the calibrated ranges, with the felling date of 50 ± 5 years, since it yields an older age compared to the other three samples (Fig. 3). We applied the same Bayesian approach in a separate model including the 14C dates from the tablets located in St. Petersburg and Berlin within the Supplementary Information (Note 3). This decision stems from concerns about the pretreatment methods, particularly in the St. Petersburg samples, where the lack of detailed documentation raises uncertainties. The use of less-stringent pretreatment procedures in these cases may potentially introduce younger contamination from conservation materials. However, the results for the Boundary start and end remain identical at 95.4% probability.

**Table 2:** The Unmodeled and Modeled ages of the four tablets. The unmodelled calibration ranges and the calibration ranges adding the estimated felling date of the tree. The calibration ranges produced in the model. All of them are obtained using the South Hemisphere calibration curve SHCal20 in OxCal 4.4 program^9,16^.

Rongorongo phase	Codes of BRAVHO lab and the ASMS lab, radiocarbon date and + N (felling date)	Unmodelled (BC/AD)				Modelled (BC/AD)			
Indices Amodel 70.2 Aoverall 71.1		From	To	From	To	From	To	From	To
		68.30%		95.40%		68.30%		95.40%	
Boundary end						1766	1869	1755	1869
Rongorongo duration						1722	1843	1712	1865
Boundary start						1693	1730	1693	1867
End boundary (between 1675 and 1869)						1766	1869	1755	1869
Last event						1754	1870	1745	1870
First event						1710	1767	1698	1865
Tablet C (Mamari) P004	BRA-6129-ETH-125845.1.1 R_Date(189;13)	1675	1810	1671	…	1680	1811	1675	1845
	BRA-6129-ETH-125845.1.1 + N(25;5)	1694	1840	1692	…	1706	1838	1699	1870
Tablet B (Aruku Kurenga) P002	BRA-6126-ETH-125843.1.1 R_Date(125;13)	1714	1924	1700	1944	1710	1831	1698	1839
	BRA-6126-ETH-125843.1.1 + N(20;5)	1732	1947	1718	1976	1727	1852	1718	1865
Tablet A (Tahua) P001	BRA-6125-ETH-125842.1.1 R Date(122;13)	1815	1923	1703	1929	1704	1721	1696	1825
	BRA-6125-ETH-125842.1.1 + N(50;5)	1862	1977	1749	1994	1752	1773	1744	1870
Start boundary (between 1675 and 1869)						1693	1730	1693	1867
Rongorongo not in the model									
Tablet D (Échancrée) P003	P003-BRA-6127-ETH-125844.1.1 R Date(474;13)	1446	1457	1438	1462				
	BRA-6127-ETH-125844.1.1 + N(50;5)	1493	1509	1483	1520				

We inserted the Date command in OxCal to give the duration of the Rongorongo phase. We constrained the start and end boundaries to 1694–1869 cal AD. Following the approach suggested by 10, the initiation date (1694 cal AD) is determined by the oldest calibrated age and the relative felling year within the range of 1694–1855 cal AD for the P004 tablet C Mamari (BRA-6129). Conversely, the termination date (1869 AD) corresponds to the date when tablet D Échancrée was sent to the Bishop of Tahiti in 1869 (Collection date). In addition, to identify the date of the earliest or latest of them we performed First and Last CQL commands in OxCal taking a sample of whichever event parameter has the minimum or maximum values (Fig. 3 and Table 2). The OxCal codes are given in the Supplementary.

The duration of the Rongorongo phase is between 1722 and 1843 cal AD (within 68.3% probability), spanning 121 years. In addition, we identified the first range of the event, between 1710 and 1767 cal AD (68.3%) and the last one between 1754 and 1870 cal AD (68.3%) (Table 2). Following the indication of Wieczorek et al.^10^ we can thus say that three of the Rongorongo tablets can be dated between 1785 and 1848 cal AD (68.3%), or even in two different times, one between the 1714–1828 cal AD and a second one between 1816 and 1840 cal AD.

The calibrated ranges of the single older date are much older than the Rongorongo phase we built, spanning between 1493 and 1509 cal AD (68.3%) (Table 2 and Fig. 3), as discussed above. The chronology of tablet D Échancrée suggests a much earlier horizon, that antedates by centuries the influx of possible external stimuli. Additionally, the non-indigenous wood used in crafting this tablet raises intriguing possibilities—perhaps sourced from distant lands as driftwood or obtained from European vessels, meticulously preserved, and revered for its utility in writing (further details on wood felling year provided in the “Method” section). The driftwood hypothesis gains significant traction, firmly anchored in the ongoing discussion surrounding the taxonomic identification of *Podocarpus sp.* (cf *Latifolia*) by Orliac^17^. Indeed, Orliac's meticulous research underscores the lingering uncertainty in pinpointing the exact species, particularly *Podocarpus latifolia*. The investigation of eight *kohau* Rongorongo tablets, including tablet D Échancrée, in her subsequent publication^12^, showed a composition featuring six tablets from *Thespesia populnea* and four from *Podocarpus sp.* (cf. *Latifolia*).

This intricate scenario opens up the intriguing possibility of an alternative *Podocarpus sp*., potentially indigenous to South America, contributing to the composition of the tablet's wood. However, when considering the wood species of the tablets under scrutiny, a noteworthy pattern emerges—all three wood types (*Fraxinus sp*., *Podocarpus sp*., *Thespesia pop*.) are renowned as preferred materials in shipbuilding, including the use of ash for ship's rudders or barrels employed for transporting goods on ships. *Podocarpus*, highly prized for ship masts, was widely employed in shipbuilding in various maritime contexts. Hence, the likelihood that the *Fraxinus* and *Podocarpus* wood, used for two of the tablets, originated from numerous European ships navigating the world's oceans since the Middle Ages and potentially reaching Easter Island by 1720, is very high.

An additional factor that warrants consideration is the "old wood" predicament. Orliac^17^ underscores the cultural and symbolic importance of *Thespesia populnea*, known as the 'rosewood of Oceania,' in eastern Polynesia, where it holds revered status as a sacred tree. Despite its sporadic mention in Rapa Nui's oral traditions and songs, the utilization of *Thespesia populnea* for the tablets resonates with its sacred nature. The scarcity of trees and the revered status of *Thespesia populnea*, recognized as the 'rosewood of Oceania,' have prompted the inhabitants of Rapa Nui to embrace a longstanding tradition of wood reuse^17^. It is plausible that the tablets were crafted from repurposed wood due to the island's tree scarcity.

Due to this scarcity and the Rapa Nui's practice of wood reuse, this custom likely extended to the tablets, particularly those manufactured from ash and *Podocarpus* wood, as these tree species were not native to the island. The tree species *Thespesia populnea* might have been introduced with the initial Polynesian settlers and persisted on the island since then. However, as discussed by Orliac^17^ (and references therein), it is noteworthy that at the arrival of the first Europeans they described the island as mostly deforested. As a result, substantial wooden pieces, such as those needed for tablets B (Aruku Kurenga), and D (Échancrée), likely predate 1720 or were fashioned from the wood of former tree trunks, existing timber, or aged wood. Consequently, the wood's age may not necessarily align with the accurate timeline of tablet manufacturing or subsequent writing. Even with the potential limited preservation of wood in the humid tropical conditions of the island, reused wood from timber, furniture, ships, or ritual objects could be preserved for some time. Thus we cannot discount that the inscription on the tablets may be more recent than the wood from which they are made. Hence, the wood's age or the felling year of the tree primarily serves as a 'terminus post quem' for dating the tablet's manufacture and, subsequently, its engraving.

Given that the tablets A (Tahua) and D (Échancrée) are crafted from wood derived from non-native tree species, it is evident that they were fashioned from repurposed wood, thus limiting their utility in dating the tablet's production and the carvings adorning them. In contrast, the significance of tablets B (Aruku Kurenga) and C (Mamari) emerges prominently. Crafted from the indigenous wood species *Thespesia populnea*, as detailed by Orliac^17^, these tablets were meticulously manufactured from trunks with a minimum diameter of 19.5 cm and a height of 15 m. Considering the island's depiction as 'treeless' in the eighteenth century when the first Europeans arrived in 1720, Orliac posits an older age for these timbers^17^. Moreover, *Thespesia populnea* is not known for its longevity, with a lifespan of around 80 years. However, we estimate a minimum of 40 annual rings in the wood of each of both tablets (Supplementary Table 2). This minimal margin of error further enhances the significance of these tablets in dating the objects accurately.

While tablet D (Échancrée) stands out as an outlier in our chronological model, displaying an older age predating the arrival of Europeans, the remaining three tablets (A, Tahua; B, Aruku Kurenga; and C, Mamari) consistently point to a main period of use in the nineteenth century, based on posterior estimates. Despite Tablet D's unique age, which challenges the prevailing chronological pattern observed in the other tablets, its distinctiveness prompts careful consideration, and further research is necessary to understand its significance in the context of the broader timeline. However, given the circumstances of preservation, we cannot assume a much later date of engraving than that in which the tree used for the tablet was cut. Our dates confirm for three (Tablet A, B, and C) of the four tablets a scenario in which the local population wrote down their texts on wood boards coming from external sources in a period that post-dates the arrival of the Europeans.

This, however, does not imply that the script they used was not invented. Indeed, the glyphs differ from any known script and, even in terms of inventory, they have no close parallels. If Tablet D's exceptional age indicates that the local population of Rapa Nui could have invented a writing system without influence or input from external agents, Rongorongo could represent one of the few independent inventions of writing in human history, adding a layer of complexity to the narrative of the cultural and historical development of the Rapa Nui inhabitants.

## Methods

### 3D modeling

To create the 3D models, we used well-established geomatic techniques, namely close-range photogrammetry, and structured light (SL) scanning, and we produced *measurable* 3D digital replicas—digital twins—of the objects with sub-millimetric geometric precision, high resolution, and high-quality texture restitution^18,19^. The downstream applications of such detailed and accurate 3D products are countless: from the digital preservation of the objects, to the update of traditional sources and transcriptions^20,21^, to the precise measurement of sign depth (allowing paleographers to disambiguate between intentional carvings and random scratches^22^), and to the extraction of orthomosaics (a 2D yet metric product with a uniform scale that allows for the accurate measurement of an object’s proportions and dimensions, just as in a map)^22,23^.

To produce the 3D models of tablets B, C, and D, we employed the methodology described in Refs.^21,22,24^, which integrates SL scanning and close-range photogrammetry. For each of the three objects, the SL 3D model and the photogrammetric one were firstly co-registered and then the texture was transferred from the photogrammetric 3D model to the mesh of the SL one using the ‘texture baking’ process^21,22,24^, producing the final 3D models of texts B, C, and D.

For tablet A, due to its large dimensions—which were not suitable for the SL scanner—we relied exclusively on photogrammetry, using the same procedure employed to produce the photogrammetric 3D models of the other three tablets, but taking care to have a higher redundancy of images (see Supplementary Table 1 for further details). More details about the 3D data collection and the processing procedure are provided in Refs.^21,22,24^.

### Radiocarbon

The procedure used for sampling was developed to be as less invasive as possible due to the importance of the pieces. Therefore, the material was collected from each table by drilling from the outside to the inside. We were able to obtain between 40 and 74 mg of wood powder that was used for the radiocarbon pretreatment. The extraction of cellulose for radiocarbon dating was performed following Cercatillo et al.^15^ with some adjustments because of the powder material and the small amount collected. In particular we did not perform the initial long (overnight) bath in NaOH and all procedures were carried out at room temperature with the exception of the oxidation step. The chemical steps involved in cellulose extraction use HCl solution to remove contamination by carbonate and to prevent CO_2_ absorption from the air. The use of a strong base as NaOH serves to remove humic acids and start the disruption of lignin structure. Lastly, an oxidation step (bleaching) follows to remove lignin, hemicellulose and other extractives. At the end of each extraction, cellulose was put in the oven at 80 °C until completely dry. An aliquot of dried cellulose equal to 2.5–3 mg was weighed into 6 × 4 mm aluminum capsules suitable for the graphitization step. The caps were previously cleaned with dichloromethane and dried at ca. 300 °C in the oven. The quantity of cellulose weighted is suitable for samples containing ca. 40% of carbon.

Once filled up with cellulose, the caps were folded and rolled up. To proceed with the graphitization process, they were combusted in the Elemental vario ISOTOPE select coupled to the AGE 3 (Automated Graphitization Equipment, IonPlusAG, Switzerland)^25^ to produce CO_2_. This gas was then reduced with an H2 injection and 3.5 mg of iron powder were used as a catalyst to produce graphite. The graphite was then pressed into a target, ready to be measured by the ETH-MICADAS Accelerator Mass Spectrometer (AMS) in Zurich. Aluminum caps with Oxalic acid II (OxII) and Phthalic acid anhydride were also prepared, combusted in the elemental analyzer and then analyzed in the AMS machine. OxII is the Radiocarbon Standard of known activity, which refers to the atmospheric activity in 1950 corrected for human activities^26^. Phtalic acid is a chemical blank and thus ^14^C-free. The radiocarbon dates are calibrated with SHcal20 in the OxCal 4.4 program^16^.

### Estimation of felling date

To date the tablets precisely by ^14^C-AMS, it is crucial to determine the position of the ^14^C-sample within the tablet, because only the outermost tree ring, under the bark, indicates the exact felling year of the tree from which the tablet was made. The inner part of the tree can be significantly older, depending on the wood species and the annual increment. Therefore, we estimated the temporal distance (number of annual increments) from the AMS sample position to the outermost preserved tree-ring. Tablet A is from *Fraxinus* wood, which forms clear and distinct annual rings that can be identified and counted on the tablet. For tablets B, C, and D, that are from *Thespesia* and *Podocarpus* wood, no distinct tree-rings could be identified. For these tablets we therefore calculated the temporal difference, that is the number of annual increments between the AMS sample position and the outermost (youngest) tree ring, using an estimated annual increment of 5 mm/a for *Thespesia* (tablets B and C) and 2 mm/a for *Podocarpus* (tablet D). The calculated temporal distances (years) were considered in the ^14^C-calibration age ranges (Table 2, Supplementary Table 2). For more details on the trees of the four tablets, see Supplementary Note 4.

### Supplementary Information

Supplementary Information.

## Data Availability

The datasets used and/or analysed during the current study available from the corresponding author on reasonable request. All data generated or analysed during this study are included in this published article [and its supplementary information files]. The data that support the findings of this study are available in the INSCRIBE ERC website https://www.inscribercproject.com/3d_viewer_home.php but restrictions apply to the availability of these data, which were used under license for the current study, and so are not publicly available. Data are however available from the authors upon reasonable request and with permission.
