# Supplementary material for: The invention of writing on Rapa Nui (Easter Island). New radiocarbon dates on the Rongorongo script
Source: Sci Rep. 2024 Feb 2;14:2794. doi: 10.1038/s41598-024-53063-7 (PMC10837134; doi:10.1038/s41598-024-53063-7)
Supplement: Supplementary file 1 — Supplementary Information. [file 41598_2024_53063_MOESM1_ESM.docx]

**Supplementary Materials for**

**The invention of writing on Rapa Nui (Easter Island). New radiocarbon dates on the Rongorongo script**

Silvia Ferrara, Laura Tassoni, Bernd Kromer, Lukas Wacker, Michael Friedrich, Francesca Tonini, Lorenzo Lastilla, Roberta Ravanelli, Sahra Talamo

Corresponding authors: S.F. (s.ferrara@unibo.it), S.T. (sahra.talamo@unibo.it)

This PDF file includes:

**Supplementary Text:** from Supplementary Note 1 to Note 6

**Supplementary Figures:** from Figure 1 to Figure 6

**Supplementary Table:** from Supplementary Table 1 to Table 3

**Supplementary Note 1 – The tablets analyzed**

In the standard corpora these tablets are cataloged as texts A-D (Supplementary Fig. 1).

A. *Tahua* (sample no. P001). This tablet presents a long text on an object shaped as an oar blade, and indeed the Rapa Nui word tahu’a means ‘house/pillar ’ or ‘house floor’, although in 1800s orthography the name of the tablet was spelled Tahua. The tablet measures 91.2 × 11.5 × 2.8 cm in size. It is made of *Fraxinus excelsior* which never grew on the island. The piece is well preserved, with no apparent traces of fire, insect, or rodent damage.

B. *Aruku Kurenga* (sample. no. P002). This object presents a dozen lines of text on both sides, it measures 41.5 × 15.2 × 3.1 cm. Its wood is Pacific rosewood (*Thespesia populnea*), which once grew on the island. It is masterfully carved; the signs are in excellent condition.

C. *Mamari* (sample no. P004). The word means ‘egg’ in Rapa Nui, which describes the shape of the tablet. It measures 29.0 × 19.4 × 2.3 cm, and it is neatly inscribed on both sides. The wood is again *Thespesia populnea*. It is well-preserved, but for some areas where the wood is damaged and shows some defects. The inscription was engraved after the damage to the surface, as some signs are carved inside the cavity. A suspension hole is observable, produced before it was inscribed. No damage from animals or fire.

D. *Échancrée* (sample no. P003). This tablet is the most damaged of the ones in the Rome collection. It measures 23.9 × 12.3 × 2.4 cm. A braid of plaited human hair was wrapped around it^1^. It became known as the ‘notched’ tablet, hence the name. The wood is identified as *Podocarpus latifolia*^2^, which never grew on the island. Other tablets are made of the same wood (tablets labeled as N, P, S). It appears unlikely that this type of wood reached the island on different occasions, which may suggest that tablets N, P, and S could be contemporaneous to D.

**Supplementary Note 2 – 3D models**

To produce the 3D models of texts B, C, and D, we employed the methodology described in ^3,4,5^, which integrates SL scanning and close-range photogrammetry. In particular, the ScanRider 1.2 SL scanner by V-GER^6^ was used to reconstruct a precise, high-resolution 3D model of the geometry of each tablet. The back dual wide camera of the iPad Pro 2020 12.9” was employed to collect the images required to produce the respective photogrammetric 3D models, consisting of high-quality texture^13^.

Considering the dimensions and the richness in details of the objects of interest, the intermediate scanning volume of the scanner^6^ was employed to find a balance between overall and local accuracy and to avoid capturing an excessive amount of data. After the initial calibration of the scanner, each object was acquired in several scans (the number increasing with the surface of the object, see Supplementary Table 1); the optical parameters of the device were adjusted and optimized from time to time, to perfectly suit the configuration of the object with respect to the optical system composed by an industrial black and white camera and a digital light projector ^7,4,5^. Moreover, for texts B and C, due to their particularly thin edges, specific markers made of small pieces of wood – placed on the surface of the objects during acquisition – were used to guide the alignment of the scans^7^. For all the three tablets, the raw scans were firstly co-registered and then cleaned, fused, and smoothed within the SpaceRider software, producing the high-resolution SL 3D models of the objects. The estimated precision of these 3D models amounts to a few hundredths of millimeters, as shown by the assessment performed on the aligned scans of tablet C and in agreement with the claimed nominal standard deviation of the scanner^7^.

The Agisoft Metashape photogrammetric software^8^, based on the Structure from Motion algorithm, was used to process the images captured with the iPad camera. For each object, several images (the number increasing with the surface of the object, see Supplementary Table 1) were collected moving the camera around the subject and framing the scene from different, but partially overlapping views. Furthermore, several scale bars were added to the setup for scaling and validating the photogrammetric 3D models with the Hold-Out Validation method^9^. The images were automatically masked to facilitate their alignment, which was performed limiting the search for key points only to the unmasked pixels^4^. The alignment was then optimized by automatically removing the uncertain tie points and refining the estimation of the camera model parameters on the remaining tie points. At the end of the alignment stage, a sparse point cloud was obtained, which was then densified. Starting from the dense cloud, the mesh and the texture were finally generated, creating the photogrammetric 3D models of texts B, C, and D.

For each of the three objects, the SL 3D model and the photogrammetric one were firstly co-registered and then the texture was transferred from the photogrammetric 3D model to the mesh of the SL one using the ‘texture baking’ process^3,4,5^, producing the final 3D models of texts B, C, and D.

For text A, due to its large dimensions – not suitable for the ScanRider scanner – we relied exclusively on photogrammetry, using the same procedure employed to produce the photogrammetric 3D models of the other three tablets, but taking care to have a higher redundancy of images (Supplementary Table 1). The estimated accuracy of the 3D model of the text A, evaluated on the Root Mean Squared Errors of the check bars, is at the level of a tenth of a millimeter. More details about the 3D data collection and the processing procedure are provided in^7,5,4^.

**Supplementary Note 3 – Previous radiocarbon dates and the new radiocarbon analysis**

All our tablets were directly radiocarbon dates and are calibrated using SHcal20^10^ in the OxCal 4.4 program^11^, using the respective felling date (Supplementary Fig. 1-5 and Supplementary Table 2 and 3).

Two other tablets were directly dated in other institutions, the Berlin Rongorongo tablet, published in^12^, and the St. Petersburg tablet published in^13^. The extraction of cellulose from the two different tablets followed the procedures described in^12,13^. The radiocarbon results were 117±14 ^14^C BP and 80±40 ^14^C BP respectively. The Berlin date produced two main different percentages of probability within the 68.3% already described in^12^. They concluded that knowing that the production of the tablets ended after AD 1870 (or even earlier after AD 1862), thus the most parsimonious conclusion is that the Berlin tablet was created sometime between AD 1706 and AD 1870, but most likely in the nineteenth century.

We incorporated these two dates into the Bayesian model (Supplementary Fig. 6 and Supplementary Table 3), which had already been constructed in the main text. The inclusion of these additional dates has resulted in a more constrained Bayesian model, reducing the span of the Rongorongo duration from 163 years to only 43 years. Notably, the results for the Boundary start and end remain identical with a 95.4% probability.

**Supplementary Note 4 – Details on the estimation of the felling years**

A. *Tahua* (sample no. P001)

Species: *Fraxinus cf. excelsior*^14^. Estimated total ring number preserved on the wood: 90. Position of the AMS sample: approx. 4.5 cm from inner (older) edge and 7.0 cm from the outer (younger) edge with approx. 40 tree-rings to the innermost (older) rings and approx. 50-60 rings to the outermost section. No sapwood or the original ‘waney edge’ (bark) is preserved, resp. could be detected. Therefore, from the position of the AMS sample a minimum of 50-60 rings to the outermost (youngest) ring are preserved on tablet A. Thus, a minimum of 50 years need to be added to the calibrated AMS age range that can serve as a *terminus post quem* date for the felling date of the tree and the carving that was applied later.

B. *Aruku Kurenga* (sample. no. P002)

Species: *Thespesia cf. populnea*^15^. Estimated total tree-ring number: 30-40. Position of the AMS sample: approx. 11 cm resp. 4 cm from the edges. The inner (older) or the outer (younger) section was not detectable. With an estimated annual growth rate of approx. 5 mm/year^16^, a maximum number of approx. 10-30 rings are preserved up to the edges. With *Thespesia* wood the natural outermost section (sapwood) would have a distinct light wood color that could not be detected on the tablet. Therefore, a minimum of 10-30 years (median: 20) needs to be added to the calibrated AMS age range that serves as a *terminus post quem* date for the felling date of the tree and the carving that was applied later.

C. *Mamari* (sample no. P004)

Species: *Thespesia cf. populnea*^15^. Estimated total tree-ring number: 40. Position of the AMS sample: 7 cm resp. 12 cm from the edges. The inner (older) or the outer (younger) section was not detectable. With an estimated annual growth rate of approx. 5 mm/year, a maximum number of approx. 20-30 rings are preserved up to the edges^16^. With *Thespesia* wood the natural outermost section (sapwood) would have a distinct light (yellow) wood color that could not be detected on the tablet. Therefore, a minimum of 20-30 years (median: 25) needs to be added to the calibrated AMS age range that serves as a *terminus post quem* date for the felling date of the tree and for the carving that was applied later.

D. *Échancrée* (sample no. P003)

Species: *Podocarpus cf. latifolia*^17^. Estimated total tree-ring number: 60-120. *Podocarpus* is a slowly growing species. Estimated growth rate is ~1-2 mm/year^18^. Position of the AMS sample: Approx. 5 cm, resp. 7,5 cm from the edges of the tablet. The inner (older) or the outer (younger) section was not detectable. With an estimated annual growth rate of approx. 2 mm/year, a number of 25-40 rings, with 1mm/year 50-80 rings are preserved up to outermost edges. Therefore approx. 25-80 years (median: c.50) needs to be added to the calibrated AMS age range that serves as a *terminus post quem* date for the felling date of the tree and for the carving that was applied later.

**Supplementary Note 5 – Restoration after the sample extraction**

From the point of view of a conservator/restorer, the method of radiocarbon dating is a micro-destructive invasive technique. For the Rongorongo objects, however, only very small quantities of wood were extracted and, at the end of the procedure, very small losses were present on the artifacts.

Despite their small size, it was nevertheless considered appropriate to provide an infilling intervention for the wooden support. This was carried out to prevent the holes, even if very small, from favoring the deposit of particles or the entry of insects (a highly improbable event, considering that the objects are housed in a museum). Moreover, the compensation for the losses preserves the full legibility – formal and aesthetic – of the artifacts.

The infilling was carried out by using Gamblin Pigmented Wax/Resin composed of beeswax, microcrystalline wax, Laropal® A-81 varnish and lightfast pigments. The PWR were chosen for their excellent properties: easy texturing, excellent adhesive qualities, moisture resistance, no ghosting residues, and relatively easy to reverse. They were applied only in the losses, by using a small spatula and texturing the surface for the best matching with the surrounding wooden support.

**Supplementary Note 6 –CQL Bayesian Codes:**

*Model in the main text, with only our four dates:*

Plot()

{

Outlier_Model("General",T(5),U(0,4),"t");

Curve("SHCal20","shcal20.14c");

Sequence()

{

Boundary("Start Boundary",U(1694,1869));

Phase("RongoRongo Phase")

{

R_Date("P001-BRA-6125-ETH-125842.1.1", 122, 13)+N(50,5)

{

Outlier(0.05);

};

R_Date("P002-BRA-6126-ETH-125843.1.1", 125, 13)+N(20,5)

{

Outlier(0.05);

};

R_Date("P004-BRA-6129-ETH-125845.1.1", 189, 13)+N(25,5)

{

Outlier(0.05);

};

First("First Event");

Last("Last Event");

};

Boundary("End Boundary",U(1694,1869));

};

Sequence()

{

Boundary("=Start Boundary");

Date("Rongorongo");

Boundary("=End Boundary");

};

R_Date("P003-BRA-6127-ETH-125844.1.1", 474, 13)+N(50,5)

{

color="red";

};

};*Model in the Supplementary, with two extra previously dated tablets:*

Plot()

{

Outlier_Model("General",T(5),U(0,4),"t");

Curve("SHCal20","shcal20.14c");

Sequence()

{

Boundary("Start Boundary",U(1694,1869));

Phase("RongoRongo Phase")

{

R_Date("Beta-1841112_St Petersburg", 80, 40)

{

Outlier(0.05);

};

R_Date("Wk50491_Berlin", 117, 14)

{

Outlier(0.05);

};

R_Date("P001-BRA-6125-ETH-125842.1.1", 122, 13)+N(50,5)

{

Outlier(0.05);

};

R_Date("P002-BRA-6126-ETH-125843.1.1", 125, 13)+N(20,5)

{

Outlier(0.05);

};

R_Date("P004-BRA-6129-ETH-125845.1.1", 189, 13)+N(25,5)

{

Outlier(0.05);

};

First("First Event");

Last("Last Event");

};

Boundary("End Boundary",U(1694,1869));

};

Sequence()

{

Boundary("=Start Boundary");

Date("Rongorongo");

Boundary("=End Boundary");

};

R_Date("P003-BRA-6127-ETH-125844.1.1", 474, 13)+N(50,5)

{

color="red";

};

};


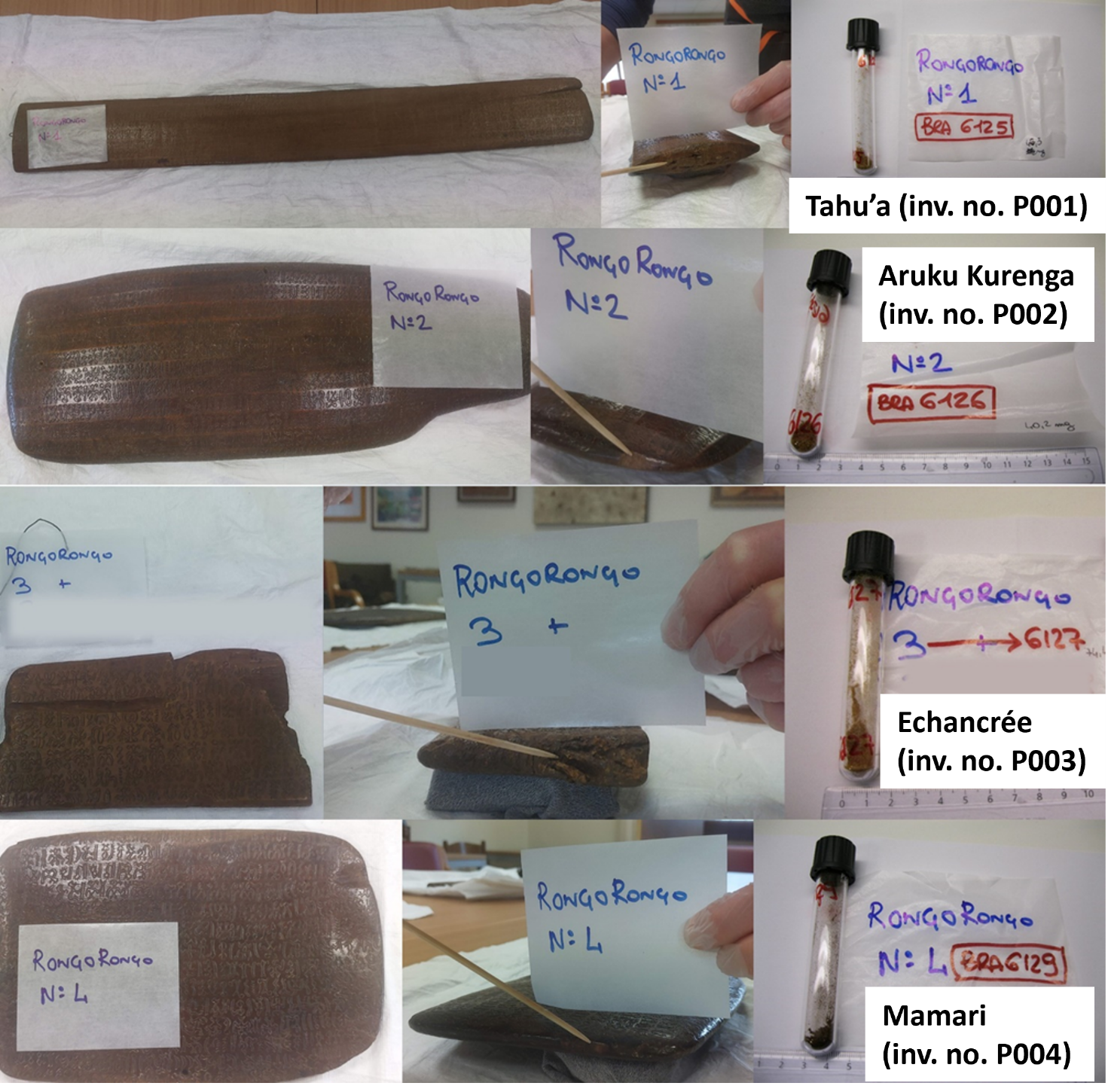


**Supplementary Figure 1.** The four tablets, with their respective code numbers, names, and indication of where the samples for ^14^C analysis were taken.

**
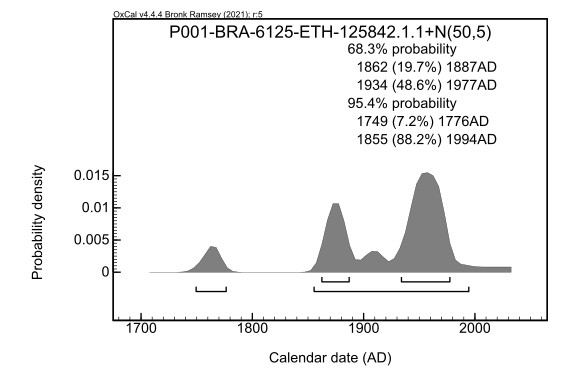
**

**Supplementary Figure 2.** Calibrated ranges with the estimated felling dated of the tree, of sample P001, tablet A Tahua (BRA-6125).

**
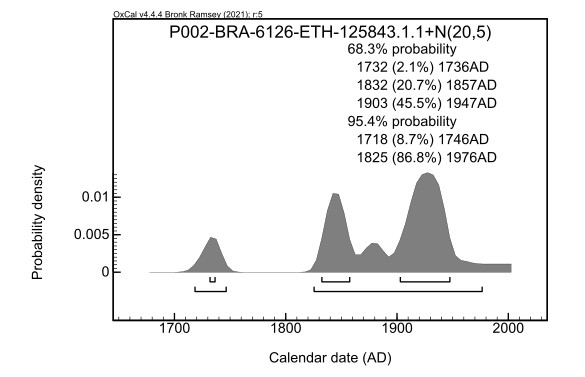
**

**Supplementary Figure 3.** Calibrated ranges with the estimated felling dated of the tree, of sample P002, tablet B Aruku Kurenga (BRA-6126).


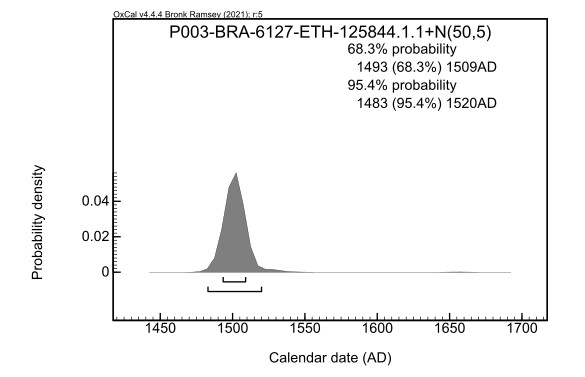


**Supplementary Figure 4.** Calibrated ranges with the estimated felling dated of the tree, of sample P003, tablet D Échancrée (BRA-6127).


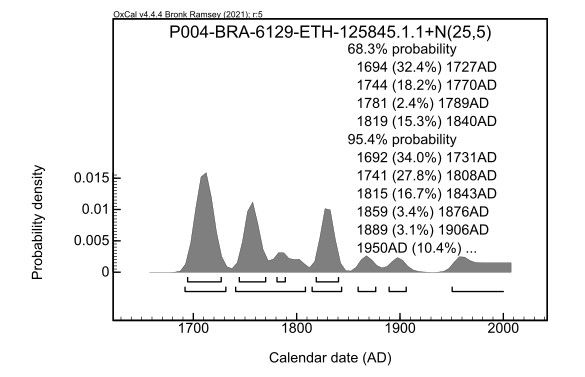


**Supplementary Figure 5.** Calibrated ranges with the estimated felling dated of the tree, of sample P004, tablet C Mamari (BRA-6129).


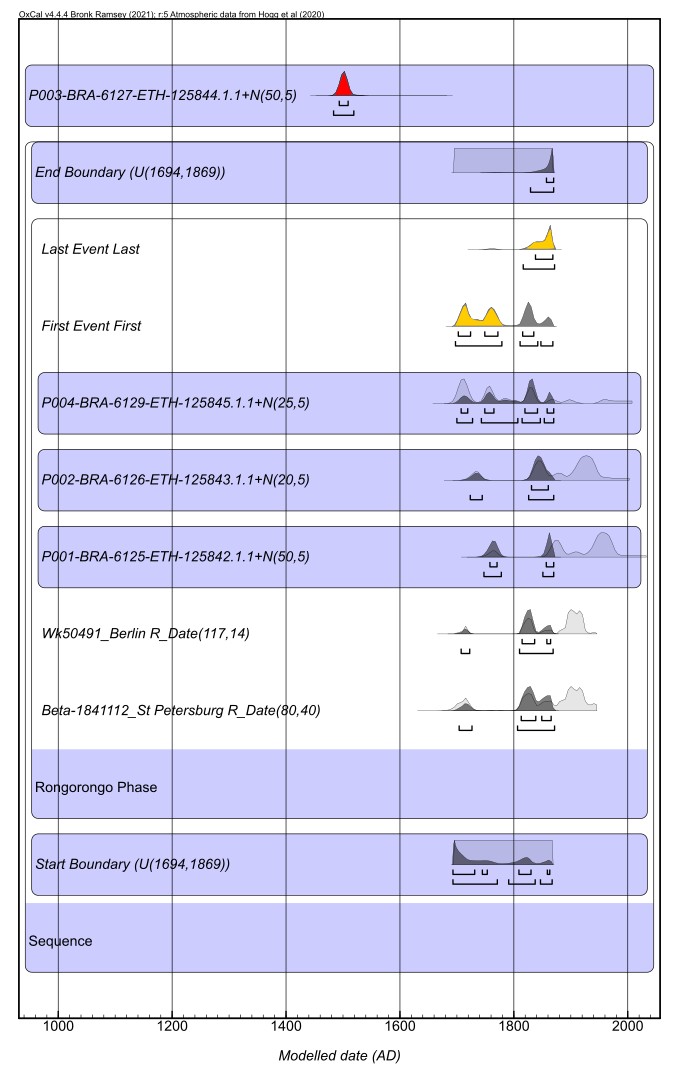


**Supplementary Figure 6.** Bayesian model with the two more dates of Berlin and St. Petersburg tablets. Bayesian model of five tablets and the calibrated age of the BRA 6127 in red. In yellow are the ranges produced in the model for the First and Last event. Radiocarbon dates were calibrated and modeled using SHcal20^11 in the main text^ in the OxCal 4.4 program^18 in the main text^

| **Object** | **3D geometry source** | **Texture source** | **No. of images** | **No. of scans** | **No. of vertices** | **No. of faces** | **Bounding box**  **dimensions** |
| --- | --- | --- | --- | --- | --- | --- | --- |
|  | [-] | [-] | [-] | [-] | [-] | [-] | [cm × cm × cm] |
| Tablet A  (Tahua) | iPad Pro 2020 12.9”  back dual wide camera | iPad Pro 2020 12.9”  back dual wide camera | 7942 | n.a. | 16253509 | 32506834 | 91.2 × 11.5 × 2.8 |
| Tablet B  (Aruku Kurenga) | ScanRider 1.2  (volume 2) | iPad Pro 2020 12.9”  back dual wide camera | 3385 | 1411 | 5527404 | 11054804 | 41.5 × 15.2 × 3.1 |
| Tablet C  (Mamari) | ScanRider 1.2  (volume 2) | iPad Pro 2020 12.9”  back dual wide camera | 3078 | 1094 | 4132332 | 8264664 | 29.0 × 19.4 × 2.3 |
| Tablet D  (Échancrée) | ScanRider 1.2  (volume 2) | iPad Pro 2020 12.9”  back dual wide camera | 462 | 330 | 9784673 | 19569346 | 23.9 × 12.3 × 2.4 |

**Supplementary Table 1.** Main features of the 3D models of the four tablets (A-D). The bounding box dimensions depend on the orientation of the 3D model with respect to its coordinate reference system (CRS): hence, they were computed after having previously re-oriented the 3D model along its principal axes through the principal component analysis (PCA) algorithm, implemented in Python.

| **Sample no. Rome** | **Object** | **Bologna AMS code** | **Species** | **Estimated total no. of rings** | **Estimated no. of rings to felling date** | **Sapwood / Waney edge** |
| --- | --- | --- | --- | --- | --- | --- |
| P001 | Tablet A Tahua | BRA-6125 | *Fraxinus cf. excelsior* | 90 | 50 | n.d. |
| P002 | Tablet B  Aruku Kurenga | BRA-6126 | *Thespesia cf. populnea* | 30-40 | 10-30 (c.20) | n.d. |
| P003 | Tablet D  Échancrée | BRA-6127 | *Podocarpus cf. latifolia.* | 60-120 | 25-80 (c.50) | n.d. |
| P004 | Tablet C  Mamari | BRA-6129 | *Thespesia cf. populnea* | 40 | 20-30 (c.25) | n.d. |

**Supplementary Table 2.** Results of the estimation of the total number of tree rings preserved on the tablets and the number of rings (years) from the position of the AMS sample to the outermost (youngest) preserved tree ring. Sapwood (which would be detectable on the wood, i.e. for the species *Thespesia*) or the natural ‘waney edge’ (bark) as an exact and reliable determination of the felling date of the trees were not detectable on any of the tablets. The age determined for the youngest tree ring obtained therefore only provides the earliest possible age for the felling date of the trees.

| **Rongorongo Phase** | **Codes of BRAVHO lab and the ASMS Lab, Radiocarbon date and + N (Felling date)** | **Unmodelled (BC/AD)** | | | | **Modelled (BC/AD)** | | | |
| --- | --- | --- | --- | --- | --- | --- | --- | --- | --- |
| **Indices  Amodel 70.2  Aoverall 71.1** |  | **from** | **to** | **from** | **to** | **from** | **to** | **from** | **to** |
|  |  | **68,30%** | | **95,40%** | | **68,30%** | | **95,40%** | |
| **Boundary End** |  |  |  |  |  | **1766** | **1869** | **1755** | **1869** |
| **Rongorongo Duration** |  |  |  |  |  | **1722** | **1843** | **1712** | **1865** |
| **Boundary Start** |  |  |  |  |  | **1693** | **1730** | **1693** | **1867** |
|  |  |  |  |  |  |  |  |  |  |
| **End Boundary (between 1675-1869)** |  |  |  |  |  | **1766** | **1869** | **1755** | **1869** |
| **Last Event** |  |  |  |  |  | **1754** | **1870** | **1745** | **1870** |
| **First Event** |  |  |  |  |  | **1710** | **1767** | **1698** | **1865** |
| Tablet C (Mamari) P004 | BRA-6129-ETH-125845.1.1 R_Date(189;13) | 1675 | 1810 | 1671 | ... | 1680 | 1811 | 1675 | 1845 |
|  | BRA-6129-ETH-125845.1.1+N(25;5) | 1694 | 1840 | 1692 | ... | 1706 | 1838 | 1699 | 1870 |
| Tablet B (Aruku Kurenga) P002 | BRA-6126-ETH-125843.1.1 R_Date(125;13) | 1714 | 1924 | 1700 | 1944 | 1710 | 1831 | 1698 | 1839 |
|  | BRA-6126-ETH-125843.1.1+N(20;5) | 1732 | 1947 | 1718 | 1976 | 1727 | 1852 | 1718 | 1865 |
| Tablet A (Tahua) P001 | BRA-6125-ETH-125842.1.1 R_Date(122;13) | 1815 | 1923 | 1703 | 1929 | 1704 | 1721 | 1696 | 1825 |
|  | BRA-6125-ETH-125842.1.1+N(50;5) | 1862 | 1977 | 1749 | 1994 | 1752 | 1773 | 1744 | 1870 |
| **Start Boundary (between 1675-1869)** |  |  |  |  |  | **1693** | **1730** | **1693** | **1867** |
|  |  |  |  |  |  |  |  |  |  |
| **Rongorongo not in the Model** |  |  |  |  |  |  |  |  |  |
| **Tablet D (Échancrée) P003** | P003-BRA-6127-ETH-125844.1.1 R_Date(474;13) | 1446 | 1457 | 1438 | 1462 |  |  |  |  |
|  | BRA-6127-ETH-125844.1.1+N(50;5) | 1493 | 1509 | 1483 | 1520 |  |  |  |  |

**Supplementary Table 3.** The Unmodeled and Modeled ages of the four tablets. The unmodelled calibration ranges and the calibration ranges adding the estimated felling dates of the tree. The calibration ranges produced in the model. All of them are obtained using the South Hemisphere calibration curve SHCal20 in OxCal 4.4 program^10,11^.

**References**

1. Horley, P. *Rongorongo. Inscribed Objects from Rapa Nui*. (Rapanui Press, 2021).

2. Orliac, C. in *The Gotland Papers: Selected Papers from the VII International Conference on Easter Island and the Pacific: Migration, Identity, and Cultural Heritage* (eds P Wallin & H Martinsson-Wallin) 125-140 (Gotland University Press, 2010).

3. Lastilla, L., R. Ravanelli, and S. Ferrara. 2019a. 3D high-quality modeling of small and complex archaeological inscribed objects: relevant issues and proposed methodology. *The International Archives of the Photogrammetry, Remote Sensing and Spatial Information Sciences*. XLII–2/W11: 699–706. doi:10.5194/isprs-archives-XLII-2-W11-699-2019.

4. Lastilla, L., Ravanelli, R., Valério, M., and Ferrara, S. 2022. Modelling the Rongorongo tablets: A new transcription of the Échancrée tablet and the foundation for decipherment attempts, *Digital Scholarship in the Humanities*, Volume 37, Issue 2, June 2022, Pages 497–516, doi:[10.1093/llc/fqab045](https://doi.org/10.1093/llc/fqab045).

5. Valério, M., Lastilla, L., and Ravanelli, R., 2022. The Rongorongo Tablet C: new technologies and conventional approaches to an undeciphered text. *Lingue e linguaggio*, *Rivista semestrale*. 2/2022, pp. 333-367, doi: 10.1418/105968.

6. V-GER. 2021. ScanRider 1.2. [www.vger.eu/brand/v-ger/scanrider-1-2](https://www.vger.eu/brand/v-ger/scanrider-1-2). (Accessed March 2023).

7. Lastilla, L., R. Ravanelli, M. Valério, and S. Ferrara. 2019b. 3D modelling of the Mamari tablet from the Rongorongo corpus: Acquisition, processing issues, and outcomes. *The International Archives of Photogrammetry, Remote Sensing and Spatial Information Sciences*, 42. XLII–2/W18: 85–89. doi:10.5194/isprs-archives-XLII-2-W18-85-2019.

8. Agisoft Metashape. 2020. Agisoft Metashape. [www.agisoft.com](https://www.agisoft.com/). (Accessed March 2023).

9. Brovelli, M.A., M. Crespi, F. Fratarcangeli, F. Giannone, and E. Realini. 2008. Accuracy assessment of high resolution satellite imagery orientation by leave-one-out method. *ISPRS Journal of Photogrammetry and Remote Sensing*, 63(4). 427–440. doi: 10.1016/j.isprsjprs.2008.01.006.

10. Hogg, A. G. et al. SHCal20 Southern Hemisphere calibration, 0–55,000 years cal BP. Radiocarbon 62, 759-778 (2020).

11. Ramsey, C. B. Bayesian analysis of radiocarbon dates. Radiocarbon 51, 337-360 (2009).

12. Wieczorek, R. M., Frankiewicz, K. E., Oskolski, A. A. & Horley, P. The Rongorongo tablet from Berlin and the time-depth of Easter Island’s writing system. The Journal of Island and Coastal Archaeology, 1-20, doi:10.1080/15564894.2021.1950874 (2021).

13. Orliac, C. The Rongorongo tablets from Easter Island: botanical identification and 14C dating. Archaeology in Oceania 40, 115-119 (2005).

14. Fischer, S. R. Rongorongo: the Easter Island script: history, traditions, texts. (Clarendon Press, 1997).

15. Orliac, C. Botanical identification of the wood of the large kohau Rongorongo tablet of St. Petersburg. Rapa Nui Journal 21, 7-10 (2007)

16. Friday, J. B., & Okano, D. (2006). Thespesia populnea (milo), ver.2.1. In C. R. Elevitch (Ed.), Species Profiles for Pacific Island Agroforestry. Permanent Agriculture Recources (PAR) (pp. 1-19). Holualoa, Hawai'i.

17. Orliac, C. in The Gotland Papers: Selected Papers from the VII International Conference on Easter Island and the Pacific: Migration, Identity, and Cultural Heritage (eds P Wallin & H Martinsson-Wallin) 125-140 (Gotland University Press, 2010).

18. Bauch, J., Quiros, L., Noldt, G., & Schmidt, P. (2006). Study on the wood anatomy, annual wood increment and intra-annual growth dynamics of Podocarpus oleifolius var. macrostachyus from Costa Rica. Journal of Applied Botany and Food Quality, 80, 19-24.
